# Multi-scale based Network and Adaptive EfficientnetB7 with ASPP: Analysis of Novel Brain Tumor Segmentation and Classification

**DOI:** 10.2174/0115734056419990250904093436

**Published:** 2025-09-15

**Authors:** Sheetal Vijay Kulkarni, S. Poornapushpakala

**Affiliations:** 1 Department of Electronics and Communication Engineering, Sathyabama Institute of Science & Technology, Chennai 600119, Tamil Nadu, India; 2 Department of Instrumentation Engineering, AISSMS Institute of Information Technology, Pune, 411001, Maharashtra, India

**Keywords:** Brain Tumor identification, Multiscale Bilateral Awareness Network, Region vision transformer-based adaptive efficientnetb7-atrous spatial pyramid pooling, MRI, MRP-HOA, Brain disorders

## Abstract

**Introduction:**

Medical imaging has undergone significant advancements with the integration of deep learning techniques, leading to enhanced accuracy in image analysis. These methods autonomously extract relevant features from medical images, thereby improving the detection and classification of various diseases. Among imaging modalities, Magnetic Resonance Imaging (MRI) is particularly valuable due to its high contrast resolution, which enables the differentiation of soft tissues, making it indispensable in the diagnosis of brain disorders. The accurate classification of brain tumors is crucial for diagnosing many neurological conditions. However, conventional classification techniques are often limited by high computational complexity and suboptimal accuracy. Motivated by these issues, an innovative model is proposed in this work for segmenting and classifying brain tumors. The research aims to develop a robust and efficient deep learning framework that can assist clinicians in making precise and early diagnoses, ultimately leading to more effective treatment planning. The proposed methodology begins with the acquisition of MRI images from standardized medical imaging databases.

**Methods:**

Subsequently, the abnormal regions from the images are segmented using the Multiscale Bilateral Awareness Network (MBANet), which incorporates multi-scale operations to enhance feature representation and image quality. A novel classification architecture then processes the segmented images, termed Region Vision Transformer-based Adaptive EfficientNetB7 with Atrous Spatial Pyramid Pooling (RVAEB7-ASPP). To optimize the performance of the classification model, hyperparameters are fine-tuned using the Modified Random Parameter-based Hippopotamus Optimization Algorithm (MRP-HOA).

**Results:**

The model's effectiveness is verified through a comprehensive experimental evaluation that utilizes various performance metrics and is compared to current state-of-the-art methods. The proposed MRP-HOA-RVAEB7-ASPP model achieves an impressive classification accuracy of 98.2%, significantly outperforming conventional approaches in brain tumor classification tasks.

**Discussion:**

The MBANet effectively performs brain tumor segmentation, while the RVAEB7-ASPP model provides reliable classification. The integration of the MRP-HOA-RVAEB7-ASPP model optimizes feature extractions and parameter tuning, leading to improved accuracy and robustness.

**Conclusion:**

The integration of advanced segmentation, adaptive feature extraction, and optimal parameter tuning enhances the reliability and accuracy of the model. This framework provides a more effective and trustworthy solution for the early detection and clinical assessment of brain tumors, leading to improved patient outcomes through timely intervention.

## 1 INTRODUCTION

Brain tumors represent a significant global health concern due to their complex pathology, high morbidity, and potential mortality. The importance of accurate and timely diagnosis lies in improving treatment outcomes and patient survival. Magnetic Resonance Imaging (MRI) is the preferred modality for detecting brain tumors, owing to its superior soft tissue contrast and non-invasive nature. However, manual interpretation of MRI scans is time-consuming, susceptible to observer variability, and limited by the complexity of tumor morphology, which includes variations in size, shape, location, and intensity. These challenges necessitate the development of automated, precise, and reliable systems for segmenting and classifying brain tumors.

Recent advances in deep learning have significantly enhanced medical image analysis, offering robust solutions for complex diagnostic tasks. Convolutional Neural Networks (CNNs) and vision transformers have demonstrated promising performance in feature extraction, pattern recognition, and image classification. Their effectiveness in analyzing brain tumors is limited by issues such as limited data availability, high computational costs, noise interference, and variability in tumor appearance. Moreover, achieving fine-grained segmentation by maintaining high classification accuracy remains a challenging task, particularly in multi-class tumor scenarios. To address these limitations, this research introduces a novel hybrid framework that combines a Multi-Scale Attention Network (MBANet) for segmentation and an Adaptive EfficientNetB7 integrated with Atrous Spatial Pyramid Pooling (ASPP) for classification. The MBANet employs a dual-path approach, incorporating both convolutional and transformer-based modules, to effectively capture fine textures and long-range dependencies. This design enables precise segmentation of tumors with irregular boundaries and heterogeneous structures.

Meanwhile, the ASPP-enhanced EfficientNetB7 classifier improves the extraction of multi-scale contextual features while suppressing noise and irrelevant patterns, resulting in improved classification performance across diverse tumor types. The proposed architecture is both effective in improving segmentation accuracy and ensuring robust classification, making it suitable for real-world clinical applications. Furthermore, adaptive optimization techniques and efficient parameter tuning are integrated to reduce overfitting and computational overhead. This work provides a comprehensive analysis of the model’s performance, evaluates its generalizability across multiple datasets, and highlights its potential as a powerful tool in computer-aided brain tumour diagnosis.

### 1.1 Brain Tumor Detection and Classification

Brain cancer is a life-threatening condition that contributes to global mortality in developed nations [1]. The human body regulates cell division under normal physiological conditions, ensuring that the growth and multiplication of existing cells result in the formation of new ones. When aged or damaged cells die, they are replaced with healthy new cells [2]. However, when this regulatory mechanism fails, abnormal or damaged cells may proliferate uncontrollably, often forming a mass of tissue known as tumours [3]. Tumors can be classified into primary or secondary types. Primary brain tumors originate within the brain and typically remain localized, whereas secondary (metastatic) brain tumors originate in other parts of the body and spread to the brain [4]. Benign tumors are non-cancerous, slow-growing masses with well-defined boundaries. Despite their rarity in attacking surrounding tissues or spreading to other organs, their size and location can cause neurological symptoms [5]. Surgical removal is the preferred treatment, often resulting in favourable prognoses with minimal recurrence [6].

In contrast, malignant tumors exhibit rapid, uncontrolled growth and aggressively infiltrate adjacent tissues, disrupting normal brain function [7, 8]. These tumors can metastasize *via* the lymphatic and circulatory systems, forming secondary growths. Clinical symptoms vary widely depending on the tumor's location and impact on surrounding neural structures [9].

### 1.2 Treatment Approaches and Diagnostic Challenges

Treatment strategies for brain tumors depend on multiple factors, including tumor type, location, patient health, and preferences [10]. Surgery aims to remove the cancer to the greatest extent, typically through a craniotomy, wherein a portion of the skull is temporarily removed to access the brain [11]. Radiation therapy is a non-invasive treatment option that utilizes high-energy radiation to target and destroy tumor cells while sparing nearby healthy tissue [12, 13]. Radioactive isotopes can also be used for internal radiotherapy. Diagnosing and treating brain tumors are complicated due to their heterogeneity at the cellular and genetic levels. Imaging challenges such as motion artifacts, variable resolutions, and inconsistencies in MRI data quality affect diagnostic accuracy [14]. Moreover, brain tumors differ significantly in size, shape, and structure, further hindering consistent detection across patient populations and tumor types [15].

### 1.3 Advancements in Deep Learning for Brain Tumor Detection

Medical imaging, particularly MRI, plays a crucial role in the identification of brain tumors. Deep learning algorithms, particularly Convolutional Neural Networks (CNNs), have shown remarkable potential in improving diagnostic accuracy [16]. By reducing false positives and negatives, these models facilitate faster and more accurate decision-making in clinical settings [17]. Recent developments highlight the use of YOLO-based deep learning frameworks for brain tumor detection. In 2024, Almufareh *et al*. demonstrated the efficacy of YOLOv5 in identifying various tumor types, including gliomas and pituitary tumors, using MRI images [18]. The model's architecture efficiently manages large datasets, improving both accuracy and computational performance. In 2023, Rajendran *et al.* proposed an advanced detection model that utilizes deep learning to segment multiple tumor regions and accurately categorize them [19]. In 2023, Jabbar *et al.* introduced preprocessing techniques, including distance image enhancement, to enhance image quality and improve classification accuracy [20]. Similarly, in 2024, Zahoor *et al.* developed a deep neural network that integrates region-based and boundary-sensitive operations, achieving superior classification results [21]. In 2024, Khan *et al.* enhanced tumor detection by applying data augmentation and denoising across multiple MRI datasets, improving generalization and robustness [22]. In 2024, Sandhiya *et al.* addressed the issue of limited labeled data by introducing synthetic image generation to facilitate training [23]. Further innovations include the ResNet-based deep learning model by Mehnatkesh *et al.,* which optimized architecture and hyperparameters to reduce manual error and improve convergence speed [24]. In 2023, Balamurugan and Gnanamanoharan introduced a Deep DCNN with an enhanced LuNet algorithm to efficiently extract and classify significant features from brain scans [25].

### 1.4 Challenges in MRI Analysis and Proposed Solutions

The accurate detection and classification of brain tumors using MRI remains a challenging task due to the variability in tumor appearance and artifacts in the images. MRI is a valuable tool for obtaining detailed anatomical information about brain tissue; however, current methods are limited by several key limitations.

#### 1.4.1 Tumor Heterogeneity

Significant variation in tumor shape, size, and texture complicates segmentation. The proposed MBANet model addresses these issues through a dual-path approach, combining texture and dependency features to achieve this.

#### 1.4.2 Image Noise

MRI noise obscures critical features needed for accurate classification. This is mitigated through EfficientNetB7, which filters out irrelevant data.

#### 1.4.3 Feature Redundancy

Existing models often rely on overlapping features, leading to inefficiency. Vision transformers in the proposed model enhance attention to relevant regions, improving classification robustness.

#### 1.4.4 Overfitting and Precision Issues

Traditional models are prone to overfitting and limited precision. The incorporation of multi-scale dilated convolutions addresses these challenges by improving generalization and tumor detection accuracy.

The interpretation of high-dimensional data generated by MRI scans can be challenging due to noise, artifacts, and inconsistencies across imaging systems. These limitations hinder the precise segmentation and classification of brain tumors. To address these issues, a novel segmentation and classification framework is required, and it offers the advantages stated below:

#### 1.4.5 Enhanced Precision and Speed

The integration of deep learning accelerates segmentation and classification, facilitating early diagnosis and improving treatment outcomes, particularly critical in emergency care settings.

#### 1.4.6 Advanced Segmentation with MBANet

This model employs a hybrid architecture that combines convolutional layers and transformer blocks to capture both fine-grained textures and long-range dependencies, thereby enhancing segmentation performance [1].

#### 1.4.7 Adaptive Classification with ASPP and RVAEB7-HOA

The ASPP module enables multi-scale feature extraction, while the optimization algorithms fine-tune classification parameters, improving precision and reducing training complexity.

#### 1.4.8 Efficient Feature Selection

EfficientNetB7 minimizes the influence of noise by emphasizing relevant features, enhancing classification accuracy.

#### 1.4.9 Noise Robustness and Redundancy Reduction

Vision transformers selectively focus on significant regions, reducing data redundancy and increasing model robustness.

#### 1.4.10 Multi-Scale Learning

The use of dilated convolutions across different scales captures features from tumors of various sizes and shapes, enhancing detection reliability.

This proposed comprehensive framework combines robust segmentation, efficient classification, and deep learning strategies to significantly advance the state of brain tumor detection using MRI, thereby enhancing clinical outcomes and supporting timely and effective treatment planning. The features and challenges of existing deep learning-based brain tumor detection and classification models are provided in Table **1**.

## 2 MATERIALS AND METHODS

### 2.1 Architectural View of Implemented Brain Tumor Segmentation and Classification Model using an Advanced Deep Learning Network

To isolate a tumor from the surrounding healthy tissue, segmentation involves splitting an MRI image into relevant areas. To accurately determine the cancer based on its size and location, this is necessary. Classification involves categorizing the segmented tumor into specific types. By effectively segmenting tumors and classifying them into distinct types, proposed models enable healthcare specialists to provide personalized care to patients. Various tumor types, such as meningiomas and gliomas, have unique features that make the segmentation process more challenging. Accurate tumor boundary segmentation can be difficult in MRI images due to noise and contrast fluctuations. Differences in imaging methods cause variations in the features and quality of images. The task of precisely segmenting a tumor, especially when it has spread to adjacent healthy tissue, is challenging. The existing tumor detection method requires a substantial amount of time for the training process. This complex model overfits the training data and performs poorly when applied to new data. Therefore, to overcome these issues, a robust model is developed that improves training datasets and provides better results. The architectural diagram of the recommended brain tumor segmentation and detection model is given in Fig. (**1**).

Anovel paradigm for brain tumor classification is developed along with a segmentation approach. In tumor analysis, the combination of a segmentation technique and a classification model improves the precision and effectiveness of treatment selection and diagnosis. The MRI images needed are gathered from a dataset that requires careful selection to ensure they are suitable for model training. The collected images undergo a segmentation process. Specialists acquire a better understanding of tumor size, form, and location in relation to adjacent structures by tracing the tumor’s borders. Accurate localization is crucial for planning radiation therapy, surgery, or other treatments while minimizing damage to healthy tissue and effectively targeting the cancer. In this proposed model, the MBANet model is designed to perform segmentation tasks. By effectively capturing both local textural details and long-range dependencies in the imaging data, this network is designed to enhance the quality of the segmented image. Segmentation provides clearer visualizations of tumors for more accurate diagnoses. Finally, the classification is done using this segmented image with the help of the RVAEB7-ASPP network. This method utilized a regional-to-local attention mechanism to effectively capture both global context and local details in the MRI images. The feature obtained from RViT is fed to RVAEB7-ASPP. The EfficientNetB7 architecture can scale efficiently while maintaining high accuracy. The pooling layer of EfficientNetB7 is replaced with ASPP, resulting in high classification precision due to the effective capture of both local and global features. This model is fine-tuned using MRP-HOA to adjust the specific attributes of MRI images. Optimization helps to maximize the precision and accuracy of this detection model and minimize FNR. The combination of RViT, EfficientNet-B7, and ASPP results in efficient classification. This model is important in medical imaging because it can handle multi-scale characteristics.

#### 2.1.1 Innovations made on the Proposed Model

The proposed model presents several notable advances in brain tumor segmentation and classification through the application of MBANet, which incorporates multi-scale procedures to refine features and preserve local image contexts, thereby enhancing the separation of the abnormal tissue. The RVAEB7-ASPP model is a new structure based on RViT with ASPP and an adaptive capability to improve the effectiveness and efficiency of EfficientNetB7, which has been used for classification. Moreover, an MRP-HOA is used to optimize the overall model performance, which is a biologically inspired metaheuristic providing the stochastic and adaptive search strategies to fine-tune the most important hyperparameters. Collectively, these developments enable improved localization to the tumor region, strong feature extraction at different scales, greater awareness of the global context of the image, and better modeling of the model across scales towards reality and generality in the analysis of images containing brain tumors.

### 2.2 Brain Tumor Dataset Collection

Dataset 1: This dataset serves as a standard data source for evaluating the performance of various classifiers. It is well-suited for training deep learning models [33].

Dataset 2: This dataset typically comprises a variety of MRI images representing various types of brain tumors. BR35H promotes the development of novel brain tumor analysis algorithms and methodologies by making its dataset publicly available. This helps to advance the field of medical imaging (https://www.kaggle.com/datasets/ahmedhamada0/ brain-tumor-detection, accessed on January 8, 2024) [34].

Dataset 3: This dataset comprises a collection of MRI images specifically focused on brain tumors. The JORDAN dataset, which is publicly accessible, stimulates experts to develop innovative methods and algorithms for analyzing brain tumors, also enhancing the field of medical imaging [2, 4, 35, 36].

The collected images from the dataset are denoted as *Ln_B_*, where the term *B* is the total amount of collected images. The sample images collected from the database are listed in the Fig. (**2**).

### 2.3 Efficient Brain Tumor Segmentation Mechanism for The Identification of Cancerous Brain Tissues

#### 2.3.1 Description of Multi-scale Bilateral Awareness Network

The traditional bilateral awareness network enhances segmentation accuracy by utilizing bilateral symmetry images. Specifically, this approach integrates the symmetric region from the images to increase the accuracy of the segmentation. However, in the proposed Multi-scale Bilateral Awareness Network, the multiscale approach is incorporated to improve the accuracy of the segmentation. The bilateral network, operating across multiple scales, is integrated to capture information from images at varying resolutions and to extract fine-grained details, yielding precise outcomes in the segmentation. Unlike the traditional bilateral awareness network, the Multi-scale module incorporated with the Bilateral Awareness Network can help better analyze global contextual information and feature representation from images to enhance the robustness of the segmentation process. The exploitation of symmetry for specific regions is predominantly focused on traditional bilateral networks. However, the proposed model accurately focuses on tumors of different sizes due to the multiscale module. A bilateral awareness network is employed in this proposed brain tumor classification framework, as it integrates both CNN and transformer models to extract the necessary information from the MRI image [26]. This network employs dual feature extraction paths, including a texture path and a dependency path, for extracting fine-grained features from the input image. Multiscale networks can identify both fine details and broader background information by capturing features at various resolutions. By analyzing images at multiple scales, this network enhances the accuracy of segmented images. Multiscale networks often employ feature fusion techniques to improve the flexibility for segmentation tasks.

#### 2.3.2 Dependency Path

It is built using the ResT-Lite architecture, which is a vision transformer model. It is specifically designed to balance the segmentation accuracy. The basic components of Res-Lite include patch embedding, stem block, and feed-forward network.

The MRI image's spatial dimensions are reduced by the stem block, and the channel dimension is increased using a series of convolutional layers, which effectively capture low-level features. Then, the features are down-sampled to generate a hierarchical feature representation using a patch embedding process. The output of each patch is formulated using (Eqs. **1** and **2**).

**Table:** 

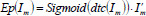	(1)

**Table:** 

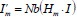	(2)

Here, the term denotes a depth-wise convolution operation. The convolutional layer is indicated as *H_m_*. The input vector is represented as *I_m_*. The dependency path consists of an efficient transformer block *E_AB_* for capturing relationships among distant pixels. The outcome of the transformer block is formulated in (Eqs. **3** and **4**).

**Table:** 

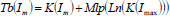	(3)

**Table:** 

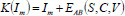	(4)

Here, the term' linear norms' is indicated, and 'multi-layer perception' is the term used. The efficient attention layer obtains vectors *S, C, V* from the input image to generate the output vector, which is provided in (Eq. **5**).

**Table:** 

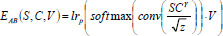	(5)

Here, the linear projection is indicated as *lr_p_*. The dimension is represented as 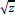.

#### 2.3.3 Texture Path

The textural data are extracted from the convolutional path to enhance the overall segmentation process. Normalization of the convolutional layer is necessary to maintain its stabilizing ability and convergence speed. The feature aggregation module combines features from both the texture path and dependency path to improve the network capacity in the segmentation task. The final segmentation map is created by combining the features from both paths. The diagrammatic representation of the multi-scale bilateral awareness network-based segmentation is given in Fig. (**3**).

### 2.4 Brain Tumor Segmentation using MBANet

The collected input image is applied to the segmentation task. Here, a Multi-scale bilateral awareness network is designed for performing segmentation tasks. Using convolutional layers, this method extracts fine-grained characteristics from MRI images. It concentrates on obtaining local information, which is essential for precise segmentation, and helps analyze the texture and margins of the tumor. Dependency Path utilizes transformer blocks to gather contextual data and establish long-range dependencies across entire images. It is crucial to comprehend the spatial relationships between the surrounding brain tissues and the tumor. The architecture handles tumor size and form fluctuations because it is made to function at several scales. In medical imaging, where tumors differ greatly in appearance, this multi-scale capacity is especially useful. A larger receptive field is made possible by the addition of transformer blocks, allowing the network to consider a wider range of contextual information, which aids in the accurate segmentation of tumors that are possibly adjacent to important brain structures. The function aggregation module combines the outputs of both the texture and dependency paths. Finally, the combined features are fed into a final prediction layer, typically a softmax layer, which generates a probability map for each pixel in the input image. Finally, the segmented image is indicated as *Hk^seg^_X_*, where the term *_X_* is shown as the total count of the segmented image.

#### 2.4.1 Performance Differentiation of MBANet-based Segmentation over Conventional Bilateral Awareness Network

Compared to the traditional bilateral awareness network, MBANet offers significant benefits due to its utilization of multi-scale operations to obtain both low- and high-frequency characteristics of tumors across multiple tumor sizes, as well as the accurate calculation of tumor boundaries.

- The benefit of this multi-scale processing is that spatial context awareness is also increased, enabling the processing of complex spatial relationships and textures with greater ease, which is another important area in segmenting brain tumors correctly.
- In the enhancement of multi-scale and bilateral components within MBANet, the preservation capability of image details and edges is improved, allowing the network to perform more accurate segmentation than previously introduced in homogeneous image parts, which commonly results in a loss of boundary sharpness in conventional bilateral-based networks.
- Additionally, MBANet has been proven to be more robust to noise and intensity variations due to the integration of information across scales, which is a beneficial aspect in terms of real-world clinical applications.
- Through the incorporation of a robust feature fusion model and support for long-range dependency acquisition, MBANet produces a set of more discriminative features to identify tumors and healthy tissue, and improves the accuracy of segmentation even higher than that of classical bilateral networks.

The segmented outcomes of the proposed MBANet model are provided in Fig. (**4**).

### 2.5 Explanation of Proposed MRP-HOA

In this proposed brain tumor classification model, the attributes of EfficientNetB7 are tuned using the designed MRP-HOA strategy. The purpose of this algorithm is to identify the best solutions by mimicking the behavior of hippopotamuses when traversing their surroundings, especially in rivers and ponds. To keep the optimization process robust, MRP-HOA incorporates procedures that mimic hippopotamuses' defensive actions against dangers. To improve its capacity to explore the solution space efficiently, this algorithm additionally incorporates strategies for avoiding predators.

#### 2.5.1 Novelty

Similar to several optimization techniques, the conventional HOA performance varies depending on the parameters selected [27]. It is difficult to determine the best values for these factors, which cause errors. Depending on the size and complexity, the performance of HOA varies. As the optimization issue becomes more complex, the technique's effectiveness and precision may decrease. Despite being designed to prevent local minima, the HOA still faces the risk of converging prematurely to poor solutions, particularly in multimodal or very complex environments. These drawbacks are reduced by modifying the random variable *Gr* used in (Eq. **6**) of the conventional HOA.

The mathematical expression of updating position in HOA is given in (Eq. **6**).

**Table:** 

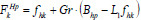	(6)

Here, the term *F^Hp^_k_* is the current position of the individual, and *B_hp_* is the position of the dominant member, *L*_1_ which is the constant integer that varies from [1-2]. The position of the hippopotamus is represented as ƒ*_hk_*. The term *Gr* is the random variable varies from [0-1]. The term *Gr* is updated based on its fitness function, and the modified concept is provided in (Eq. **7**).

**Table:** 

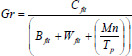	(7)

Here, the present fitness value is indicated as *C_fit_* the best fitness value *B_fit_*, the mean value is *Mn*, and the worst fitness value is *W_fit_*. The total population is displayed as *T_p_*. Modifying random variables enhances the range of potential solutions. This modification enables MRP-HOA to explore different portions of the solution space and is essential for preserving a good exploration-exploitation balance. Depending on the fitness value, MRP-HOA dynamically modifies its search plan. By randomly modifying solutions and focusing on regions with higher fitness ratings, MRP-HOA aims to explore surrounding areas and produce better results. The pseudocode of the modified MRP-HOA is described in Algorithm **1.**

**Algorithm 1:** Designed MRP-HOA.

**Input: Attributes for optimization**	
Iteration count and population size are established.	
The current position is selected.	
The best position is identified based on its fitness function.	
**Modify the random variable *Gr* using Eq. (7)**	
For *F* → 1 *to T_max_*	
For *k* → 1 *to T_p_*	
**Phase 1: Exploration stage**	
Calculate the new position.	
Update the new spot using Eq. (**6**)	
**Phase 2: Exploitation phase**	
The new boundary of variable decision is evaluated.	
End for	
End for	
**Output: Optimized attributes**	

### 2.6 Deep Learning Aided Classification Mechanism for Distinguishing Brain Cancer Tissues for Better Diagnosis

Layer Description of Region Vision Transformer: In this proposed brain tumor categorization model, the RViT model is developed to perform classification tasks. RViT considered segmented images as input, which is fed to the tokenization layer. The standard ViT is improved by introducing regional tokens for capturing the finer and local contextual details in the images. The conventional ViT considers the entire image in a fixed condition, but the proposed RViT can have the capability to create the regional tokens for a particular region of the image. The tumor boundaries in the medical images are focused by the regional tokens in the proposed RViT, and this model is used for attaining an accurate feature representation by understanding the spatial relationship within the region of the images using the regional information. In addition, the adaptability and generalization of the tumor classification are improved by the regional tokens as they handle the variation in the shape, appearance, and size of the tumor. The RViT model does not process the entire image because the regional token in this model prioritizes the important region in the image, which helps to reduce the computational process involved in the tumor classification.

#### 2.6.1 Patch Tokenization

A non-overlapping patch (*e.g*., 16x16 pixels) is created from the input image. After being flattened, each patch is projected linearly into a token representation. Through this procedure, the image is converted into a series of tokens that the transformer is able to handle. The model produces two different kinds of tokens.

- Regional Tokens: Capture the global context of the image by representing greater sections of it.
- Local Tokens: Depict more localized features and smaller patches.

#### 2.6.2 Positional Encoding Layer

Obtained positional embeddings are included in every token to preserve spatial information. This helps the model interpret the relative locations of the patches within the source image.

#### 2.6.3 Transformer Encoder Layers

Each transformer encoder consists of a self-attention mechanism that allows the model to learn relationships between tokens. Regional Self-awareness determines attention between regional tokens to gather global data, and local self-awareness enables intricate local interactions by calculating attention among local tokens linked to each regional token. The feed-forward and self-attention layers are followed by a normalization layer to stabilize and speed up training.

#### 2.6.4 Pyramid Layout

This architecture makes use of a pyramid structure, with increasingly fewer tokens in the deeper layers. This enables the model to capture characteristics at various sizes successfully.

#### 2.6.5 ASPP Layer

This layer gathers multi-scale contextual information by applying dilated convolutions at various speeds. This enables the model to extract information from different spatial resolutions, which is crucial for recognizing tumor sizes. A fixed-size representation is produced by pooling with the help of the output from the last transformer layer. Global average pooling, which averages the feature maps across spatial dimensions, is frequently used for this process.

#### 2.6.6 Fully Connected Layer

Final class scores are obtained from the pooled representation through one or more fully connected layers.

#### 2.6.7 Softmax Activation

To enable multi-class classification, a softmax function is applied to the fully connected layer's output to generate probabilities for each class.

#### 2.6.8 Output Layer Class Predictions

Based on the highest probability from the softmax layer, the model produces the predicted class labels. The diagrammatic illustration of the RViT model is provided in Fig. (**5**).

### 2.7 EfficientNetB7 with ASPP: Network Description

This novel brain tumor classification model, EfficientNetB7 with ASPP, aims to categorize different types of brain tumors.

#### 2.7.1 EfficientNet B7

It is a deep structural design designed for an image classification strategy. It uses the compound scaling method to uniformly scale certain factors like the depth *Dt*, width *Wd*, and resolution *Rs* of the network. The total number of layers in the network is measured using the scaling factor *Dt*, the number of channels in each layer is measured using the scaling factor *Wd*, and the size of the image is measured using the scaling factor *Rs*. The mathematical form of scaling factors is expressed in (Eqs. **8**-**10**).

**Table:** 

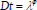	(8)

**Table:** 

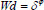	(9)

**Table:** 

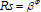	(10)

Here, the term *φ* is the compound coefficient, and (*λ*,*δ, β*) the constant values for scaling factors. This n etwork is designed to optimize the model reliability using the MBConv block. The feature map was reduced to simplify the model, making it less complex and easier to train. To improve the training ability of the network, the batch normalization process helps to standardize the input by scaling the activation of the previous layer.

For each extracted feature, the mean *ѱ* and standard deviation *σ*^2^ are calculated using (Eqs. **11** and **12**).

**Table:** 

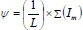	(11)

**Table:** 

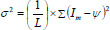	(12)

The number of instances is represented as *L*, and the output of global pooling is fed to the softmax classifier for final classification purposes, which makes it particularly effective for tasks like brain tumor classification from MRI images. The number of feature channels in EfficientNetB7 is increased by a sequence of MBConv blocks while the input is gradually downsampled.

ASPP Module: It is added after EfficientNetB7's feature extraction layers. This module is used to capture characteristics at numerous scales since it has many parallel convolutional layers with varying dilation rates *(e.g*., 1, 6, 12, and 18). ASPP consists of global average pooling to capture global context, and a layer of concatenation to combine the outputs of parallel convolutions. Finally, the output from the ASPP module is then passed through a Softmax classifier for multiclass classification tasks.

The diagrammatic representation of EfficientNetB7 with ASPP is illustrated in Fig. (**6**).

### 2.8 Brain Tumor Classification using RVAEB7-ASPP

A novel RVAEB7-ASPP model is developed for classifying the types of brain disorders, which enhances the diagnostic success rate of the proposed model. The segmented image *Hk^seg^_X_* is applied to RVAEB7-ASPP for classifying the tumor in the brain. By combining EfficientNetB7 with ASPP, the model can obtain features at different scales, which enhances its ability to identify tumors of various sizes and characteristics. RViT utilizes an attention process to generate relevant features, which are critical for essential brain tumor categorization. The entire image is processed, and long-range dependencies are captured by RViT, which results in a clear visual of the affected area in the surrounding brain tissue. It is a highly efficient CNN that balances the depth and resolution of the segmented images, leading to improved performance with fewer parameters.

Designed EfficientNetB7 is the main network to perform classification tasks. The pooling layer of EfficientNetB7 is replaced with ASPP. Both RViT and EfficientNetB7's features are incorporated into the ASPP layer to produce the final classified outcome. The attributes, like hidden neuron count, number of epochs, and activation function, are fine-tuned with the help of MRP-HOA to boost the classification accuracy with limited training data. The adaptive nature of EfficientNetB7 helps to reduce the computational complexity and overfitting issues. The combination of RViT and ASPP with EfficientNet B7 leads to improved accuracy and robustness in segmentation and detection tasks. The model influences the strengths of both components to achieve better results than using EfficientNet B7 alone. EfficientNet B7 has already been built with high accuracy. Its capabilities are improved by including RViT and ASPP without appreciably raising the proposed model complexity, which makes it appropriate for implementation in settings with limited resources. By combining these advanced elements, the designed framework generates better state-of-the-art outcomes using benchmark datasets. The mathematical expression of the objective function is provided in (Eq. **13**).

**Table:** 

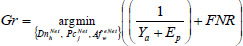	(13)

Here, the optimized hidden neuron count *Dn_h_^Net^* varies in the range of [5-225], the optimized epoch count is indicated as *Pc_j_^Net^* varies in the range of [5-50], and the optimized activation function is specified as *Aƒ_w_^eNet^*, which varies in the range of [0-4]. The accuracy and precision of the designed model are improved during the classification phase with minimum FNR. The mathematical forms of Accuracy *Y_a_* using (Eq. **14**), precision *E_p_* using (Eq. **15**), and FNR are provided in (Eq. **16**).

**Table:** 

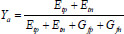	(14)

**Table:** 

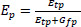	(15)

**Table:** 

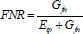	(16)

Here, the term *E_tp_* is indicated as a true positive value, *E_tn_* a true negative value, *G*_ƒ_*_p_* a false positive value, and *G*_ƒ_*_n_* a false negative value.

ASPP network employs multiple parallel Atrous convolutions with different rates to capture features at various scales, which is crucial for identifying tumors of different sizes and shapes. ASPP can obtain spatial information by pooling features at various scales and reducing dimensionality, which is beneficial for categorizing tumors as normal or abnormal. The combination of RViT and EfficientNetB7 with ASPP allows for a more robust feature extraction process, leading to higher classification precision for brain tumors. The hyperparameter details of the proposed model are given in Table **2**.

The diagrammatic specification of the RVAEB7-ASPP-based brain tumor classification process is given in Fig. (**7**).

## 3 RESULTS

### 3.1 Experimental Setup

The implementation is done using Python software. Both training and testing sets are used for implementation with chromosome length as 3, number of populations as 10, and maximum iteration as 50. The outcome of the designed scheme was assessed among dissimilar metrics, and the outcomes were compared with conventional algorithms like Tasmanian Devil Optimization (TDO), Fennec Fox Optimization (FFO), Horse Herd Optimization (HHO), and Hippopotamus Optimization Algorithm (HOA) [27-30]. Certain techniques like CNN, DCNN, YoloV5, and RAN were also utilized to compare the outcome of the proposed brain tumor classification model [18, 19, 25, 31].

### 3.2 Performance Metrics

The equations below provide the statistical expression of various performance metrics.

The F1 score is the harmonic mean of Precision and Sensitivity, which is calculated using (Eq. **17**).

**Table:** 

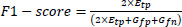	(17)

Sensitivity *S_ve_* is calculated using (Eq. **18**).

**Table:** 

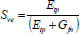	(18)

The prevalence (PT) is calculated using (Eq. **19**).

**Table:** 

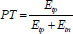	(19)

The dice coefficient *D_cf_* is estimated using (Eq. **20**).

**Table:** 

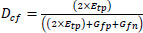	(20)

Jaccard index *J_idx_* is measured using (Eq. **21**).

**Table:** 

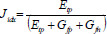	(21)

### 3.3 Convergence Analysis

Convergence analysis among algorithms is used to determine whether an iterative algorithm reaches a local or global minimum. It evaluates the speed and reliability of the algorithm's approach to the optimal solution. Understanding how quickly and efficiently an algorithm performs using training data is made easier with the aid of convergence analysis. This analysis enhances model performance by fine-tuning hyperparameters and training approaches through convergence analysis. The graphical representation of convergence analysis is provided in Fig. (**8**). The number of iterations is shown in the X coordinate. In contrast, the cost function is represented on the Y axis of the convergence analysis. The convergence of the proposed algorithms in Fig. (**8a**-**c**) is better than the conventional algorithms, and they did not fall under the condition of the local optimum. By determining the convergence rate, more effective algorithms can be chosen, which shortens the training period by allowing for faster optimization of performance. It is possible to improve patient outcomes by improving the detection accuracy and accurate diagnosis by making sure that models converge well. Advanced algorithms are implemented in healthcare settings where resources are limited by using efficient convergence to lower the computational complexities needed for model training. By using convergence analysis, the model's precision of tumor categorization is enhanced.

### 3.4 Segmentation Analysis of the Proposed Model

The segmentation analyses of the proposed brain tumor classification model among different classifiers using various datasets are provided in Fig. (**9**). The accurate delineation of tumor boundaries can be achieved through segmentation. Specialists precisely determine the location of a tumor within the MRI by using segmentation to outline the borders of the cancer. Segmentation reduces the likelihood of false positives and enhances tumor identification accuracy by separating malignancies from surrounding tissues. The segmentation process is crucial for assessing tumor growth or shrinkage over time, aiding in treatment evaluation. Segmentation provides clearer visual representations of tumors, making it easier for radiologists to interpret imaging results. The X axis in this analysis shows the models used for the segmentation process, and the Y axis shows the performance measures in percentage terms. The accuracy of the proposed MBANet-based segmentation is 98.9% higher than other existing segmentation models like Unet, Unet3+, TransUnet, and TransUnet3+. The Fig. (**9a**) indicates the respective dataset's superior performance in terms of accuracy. According to Fig. (**9b**), the proposed MBANet attained a Dice score beyond 0.85%. Likewise, the IoU of the proposed MBANet in Fig. (**9c**) also reaches an improved value compared to the other approaches. The Fig. (**9d**) specifies efficient segmentation techniques that provide a better understanding of the tumor's interaction with surrounding components. Thus, segmentation enables to define of the borders of tumors precisely and differentiates them from the surrounding healthy tissue.

### 3.5 Performance Analysis by Varying Epoch Count

Performance analysis plays a crucial role in improving brain tumor detection, particularly when varying the epoch count during the training. The graphical illustration of performance analysis among numerous algorithms and techniques across different epoch counts is provided in Fig. (**10**). Fig. (**10a**) shows F1-scores across epochs; the data at each epoch is used by this scheme to modify its weights based on predicted and real values. The learning algorithm's iteration through the training dataset is dependent upon the number of epochs. This may have a major impact on its capacity for learning and generalization. The specialist monitors key performance metrics by changing the epoch count and assists in determining the optimal number of epochs that yield the best performance without overfitting. Here, the epochs are indicated on the X axis, whereas the values of various performance metrics are shown on the Y axis. As per Fig. (**10b** and **c**), the suggested MRP-HOA-RVAEB7-ASPP reaches the PT value of 18% and sensitivity of 86% at the 400th epoch. At epoch count 100, the F1-score of the proposed MRP-HOA-RVAEB7-ASPP model is enhanced by 6.74% over CNN, 10.4% over Yolov5, 13.09% over DCNN, and 25% over RAN. Thus, the performance evaluation indicates that the deep MRP-HOA-RVAEB7-ASPP model may greatly improve the identification of brain tumors.

### 3.6 Statistical Analysis of the Proposed Model

By analyzing data patterns and model performance, statistical analysis is used to assess the performance of tumor detection. The numerical values of statistical analysis among different datasets are provided in Table **3**. Statistical measures sum up the characteristics of the dataset, including image features and tumor types. Such information makes it easier to spot outliers and abnormalities that might interfere with model training. Statistical methods are used to analyze survival data and identify factors that influence patient outcomes. By concentrating on clinically significant aspects, this analysis enhances detection accuracy. The mean value of the MRP-HOA-RVAEB7-ASPP model is decreased by 26.8%, 24.8%, 21.07%, and 10.6% than TDO-RVAEB7-ASPP, FFO-RVAEB7-ASPP, HHO-RVAEB7-ASPP, and HOA-RVAEB7-ASPP. By using statistical models to group patients according to their risk characteristics, personalized detection and treatment plans might be developed.

### 3.7 K-fold Value Evaluation of the Proposed Model among Algorithms and Techniques

Statistical method used to evaluate the consistency of the brain tumor detection model. Cross-validation minimizes the variation caused by a single train-test split by averaging the outcomes over K folds, giving a more reliable assessment of the model's performance. The numerical analysis of the recommended model based on Datasets 1 and 2 is provided in Tables **4** and **5**. Data utilization is increased for both training and validation, resulting in enhanced dataset utilization, which is especially beneficial for small datasets. The performance of various algorithms and methods is methodically compared using K-fold value variation. The best-performing model on the same dataset is determined by using the K-fold approach on many models. Based on dataset 1, the accuracy of the MRP-HOA-RVAEB7-ASPP model is 6.8% higher than TDO-RVAEB7-ASPP, 8.04% higher than FFO-RVAEB7-ASPP, 6.8% higher than HHO-RVAEB7-ASPP, and 4.4% higher than HOA-RVAEB7-ASPP. Thus, the overall detection accuracy of the proposed model is 97% at the K-fold value 2, which is highly efficient in the classification of brain tumors.

### 3.8 Dice score Analysis of the proposed model over the baseline approaches

The quantitative analysis of the dice score of the proposed MBANet over the baseline approaches is indicated in Table **6**. It is noted from Table **6** that the developed MBANet model attained the dice score of 91.4% whereas the baseline models, such as Unet, Unet3+, TransUnet, and TransUnet3+, reached the dice score of 87.3%, 88.9%, 89.6%, and 90.7% respec-tively. As compared to the baseline models, the proposed MBANet shows an improvement of 1 to 4%, which confirms the effectiveness of the proposed MBANet over the baseline approaches.

### 3.9 Quantification of the Computational of the Recommended Model

Table **7** provides various details of the proposed RVAEB7-ASPP model over the existing approaches. According to Table **7**, the inference time of the designed RVAEB7-ASPP is 0.062 sec, and the size of the model is 48MB. The lower inference time of the recommended RVAEB7-ASPP represents that it can take only a minimum time to process the input images, at the same time, it provides accurate results in the classification of the brain tumor. In addition, the lower size of the proposed RVAEB7-ASPP does not necessitate a complex hardware setup for the implementation process. The minimum interference time, hardware requirement, and FLOP of the designed RVAEB7-ASPP model are used to confirm the viability of the proposed model in the clinical environment.

### 3.10 Clinical Validation of the Recommended Model

This section discusses the clinical validation of the designed RVAEB7-ASPP. In the clinical validation, the collaboration is made with the research institution, radiology center, *etc*. This validation Table **8** is used to ensure the performance of the designed model outside the dataset. The performance of this model in the real-time diagnostic scenario is evaluated through the implementation of the developed model in ongoing clinical research. The number of participating radiologists is taken as 3. Inter-observer variability is used for measuring the agreement among the radiologists and prediction of the designed RVAEB7-ASPP, where Cohen’s kappa is used for the validation, and the proposed model attained 0.86, which represents the consistency of the proposed model relative to the healthcare specialist. The real-world applicability of the proposed model is thoroughly analyzed by performing the clinical validation with the healthcare institution and partnership, which is used for enhancing the confidence of the designed model among the healthcare experts.

## 4 DISCUSSIONS

The convergence analysis of the proposed RVAEB7-ASPP is given in Fig. (**8**) For the 1st dataset, the proposed model converges rapidly over the prior models. Fig. (**9**) illustrates the segmentation assessment of the designed MBANet in terms of various performance indices. Here, the proposed MBANet model outperforms the Unet, Unet3+, TransUnet, and TransUnet3+ with greater accuracy, while the UNet model attained lower accuracy compared to the other approaches. Fig. (**10**) indicates the epoch-based analysis of the suggested MRP-HOA-RVAEB7-ASPP for datasets 1 and 2, respectively. It can be seen from Fig. (**10a**) that the proposed MRP-HOA-RVAEB7-ASPP model attained the highest accuracy at the 500th epoch, and the HHORVAEB7-ASPP model reached the 2nd highest accuracy. Table **3** shows the statistical validation of the designed MRP-HOA-RVAEB7-ASPP. It can be seen from Table **3** that the proposed MRP-HOA-RVAEB7-ASPP reaches a higher standard deviation than the existing approaches. The K-fold-based evaluation of the suggested MRP-HOA-RVAEB7-ASPP is given in Tables **4** and **5**. The TDO-RVAEB7-ASPP and HHO-RVAEB7-ASPP achieve the lowest specificity at the first K fold, while the designed model has a specificity of 97.05%. The dice score of the suggested segmentation approach with the baseline models is given in Table **6**. As noted from the results, the proposed model attained the maximum dice score value, and here, the lower value of dice score is attained by the Unet model. The computation cost of the designed strategy over various metrics is presented in Table **6**. The designed model is used to perform brain cancer classification at a lower cost. This research introduced a multiscale deep learning model for the brain tumor segmentation and classification process. Here, the RVAEB7-ASPP is introduced for classifying the brain tumor from the segmented images, and this model provides the maximum accuracy value in the classification of brain tumors. As compared to the existing models, the proposed RVAEB7-ASPP surpassed the conventional models, including CNN, YoloV5, DCNN, and RAN, in the brain tumor classification. Thus, the results showed that the proposed RVAEB7-ASPP combines the RViT, EfficientNetB7, and ASPP effectively, enhancing the accuracy of the brain tumor classification. The prior EfficientNet B7 models only rely on the layers to provide efficient results in the tumor classification process. But here, the ASPP and RViT are combined with EfficientNet B7 to achieve a promising outcome in the classification of brain tumors. Here, the feature learning capability of the model is enhanced by the RViT incorporated in this model. The ASPP addresses the degradation problem in the EfficientNet B7 in the proposed framework. The MRP-HOA enhances the training process of RVAEB7-ASPP, which speeds up the classification process. Moreover, the MBANet model effectively segments the tumor region from the collected images. The MBANet provides precise segmentation results by strategically utilizing the multiscale information during the segmentation. The automatic extraction of brain tumors greatly increases the ability to segment brain tumor features from the images. The potential of the MBANet model is showcased by the experimental results, and it is used for automatic segmentation of brain tumors from the MRI. The RAN is proposed in to detect malignancy in the brain images of humans [31]. However, this model is computationally intensive as it did not adopt the segmentation models before the classification. The YOLOV5 model proposed by Almufareh *et al.* utilizes its complex structure for detecting the tumor regions [18]. So the proposed RVAEB7-ASPP efficient model provides robust results in the classification of brain tumors. The proposed MBANet provides a notable advancement in the segmentation process since it effectively segments the abnormal region from the collected images. However, the conventional model does not effectively segment the abnormal region from the surrounding tissues. Furthermore, this study utilizes the multiscale approach to enhance the details of the images. The importance of the deep learning model in medical imaging is emphasized by the findings of the research. The previous works demonstrate the effectiveness of the hybrid models. The computational cost of the prior models is very high since the existing models do not adopt the optimization algorithms for brain tumor segmentation and classification processes.

The proposed models precisely define tumor boundaries concerning surrounding brain structures, enhancing the understanding of the tumor's location, size, and shape. To plan surgical procedures and other treatment options, this information is crucial. The proposed model acts as a decision-support tool for healthcare professionals to develop treatment strategies. This can significantly improve the quality of care delivered to patients diagnosed with brain tumors. The proposed model combines the three modules for the classification of the brain tumor, which may enhance the computational resources in the implementation. The higher computational resources may limit the proposed model's applicability in the real-time clinical setting. The quality of the medical images greatly influences the performance of the developed model. Thus, the accuracy of the proposed model is adversely affected by the noise and artifacts in the images. The diversity of the dataset utilized in this model is limited, which greatly breaks down the generalization capability of the model [32-36].

### 4.1 Analysis of Failure Scenarios

The recommended MRP-HOA-RVAEB7-ASPP provides impressive performance in the brain tumor classification, but this model offers some misclassification results in the specified instances. This model fails to effectively analyze the typical tumor morphology and different tumor types because certain tumor slices are incorrectly labeled. The tumor with similar radiological features is sometimes misclassified by this model, which can result in a higher amount of false negatives. Due to the boundary ambiguities, the accuracy and the interpretability of the model are greatly affected since it has very low attention map clarity. The need for further improvement is raised by understanding these failure scenarios. The clinical reliability of the model will be improved by addressing these issues *via* the artifact removal approach, multimodal data, and data augmentation process. The proposed model's robustness and generalization capability will be enhanced by utilizing the large and diverse dataset in upcoming studies. In addition, advanced ensemble models will be developed in the future for automatically handling the noise and artifacts in the images used for brain tumor detection. The medical images from the other modalities will be gathered in future work for detecting the brain tumor accurately. Simplified versions of the model have been developed in upcoming studies *via* pruning and quantization for real-time use, and it has been added in future research studies.

## CONCLUSION

This paper has utilized an integrated deep learning model to segment and classify brain tumors in humans. Here, the MBANet model was best suited for segmenting the tumor region from the images, and these segmented images were classified using the MRP-HOA-RVAEB7-ASPP to take the appropriate treatment measures for reducing the morbidity of the human. Using Dataset 1, the accuracy of the MRP-HOA-RVAEB7-ASPP model is enhanced by 14.1% over CNN, 5.4% over YoloV5, 10.2% over DCNN, and 8.9% over RAN by varying the K-fold value to 1. The advanced deep learning approaches used in the HOA-RVAEB7-ASPP model help to improve performance and accuracy in segmentation and classification tasks.

### Limitations of the Proposed Model

Despite the better performance of the developed model, the suggested model faces various limitations. Potential bias issues and preconceived expectations have a significant impact on the performance of the developed model in terms of prediction. In addition, the model’s performance is not generalized well in the heterogeneous environment because of the absence of the data augmentation process. In addition, the resilience of the model is very low because of the varying quality of the images. Thus, these areas of research need further exploration to attain better performance in tumor classification.

### Future Work

As a part of the future work, the recently developed AI paradigm is named Granular-Ball Computing (GBC) for effectively capturing the heterogeneity patterns and complex structural details from the images, and it is mainly because of the global topology precedence followed by the GBC. The granular-ball neighborhood rough sets are used by the 3WC-GBNRS++ [32], which helps to enhance the classification accuracy under heterogeneous environmental conditions. Secondly, future work will integrate larger and more varied datasets with comprehensive metadata (*e.g*., tumor classifications, imaging techniques, patient demographics) and the data augmentation techniques (*e.g*., rotation, flipping, synthetic generation) to boost the generalization performance of the developed model. The resilience of the model to varying image quality can be greatly increased by combining sophisticated training techniques like adversarial training and robust loss functions with efficient preprocessing techniques. Future research should thoroughly assess these methods to guarantee reliable and consistent brain tumor classification across a range of clinical image characteristics.

## Figures and Tables

**Fig. (1):**
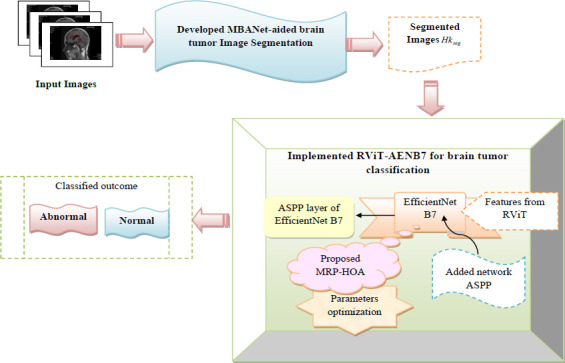
Architectural diagram of the implemented brain tumor segmentation and classification approach.

**Fig. (2):**
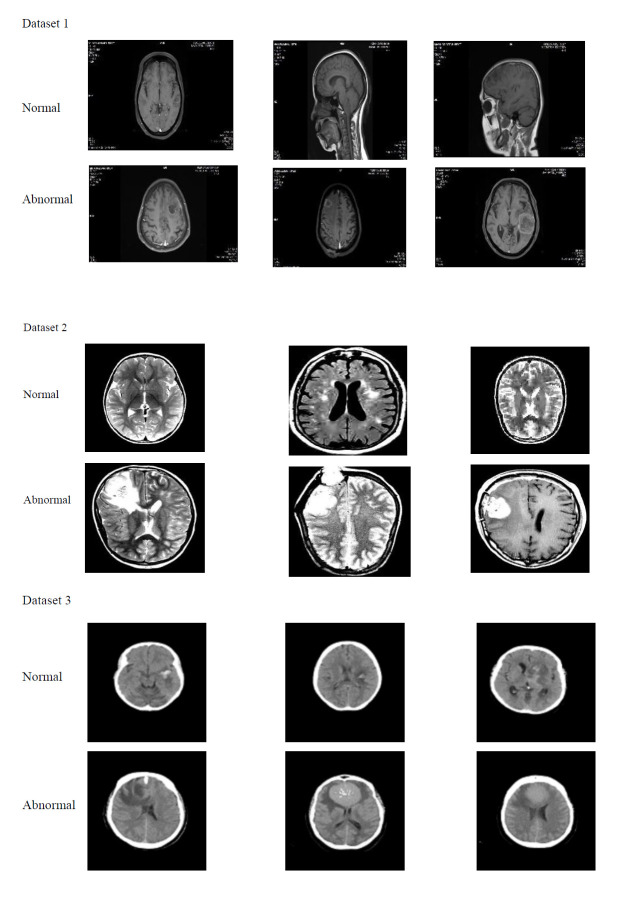
Sample images from the dataset [33-36].

**Fig. (3):**
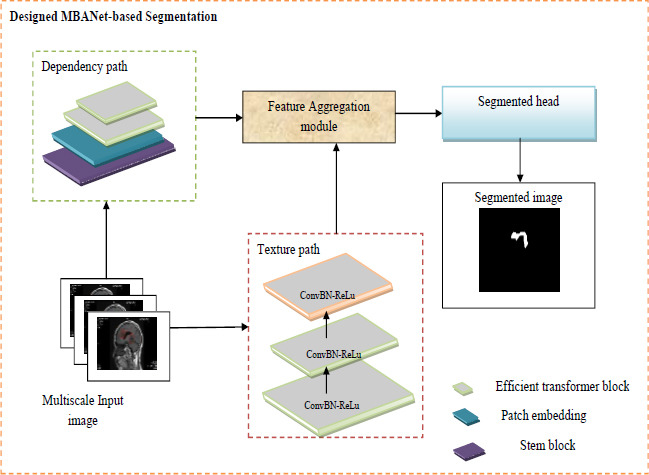
Visual illustration of the multi-scale bilateral awareness network-based segmentation.

**Fig. (4):**
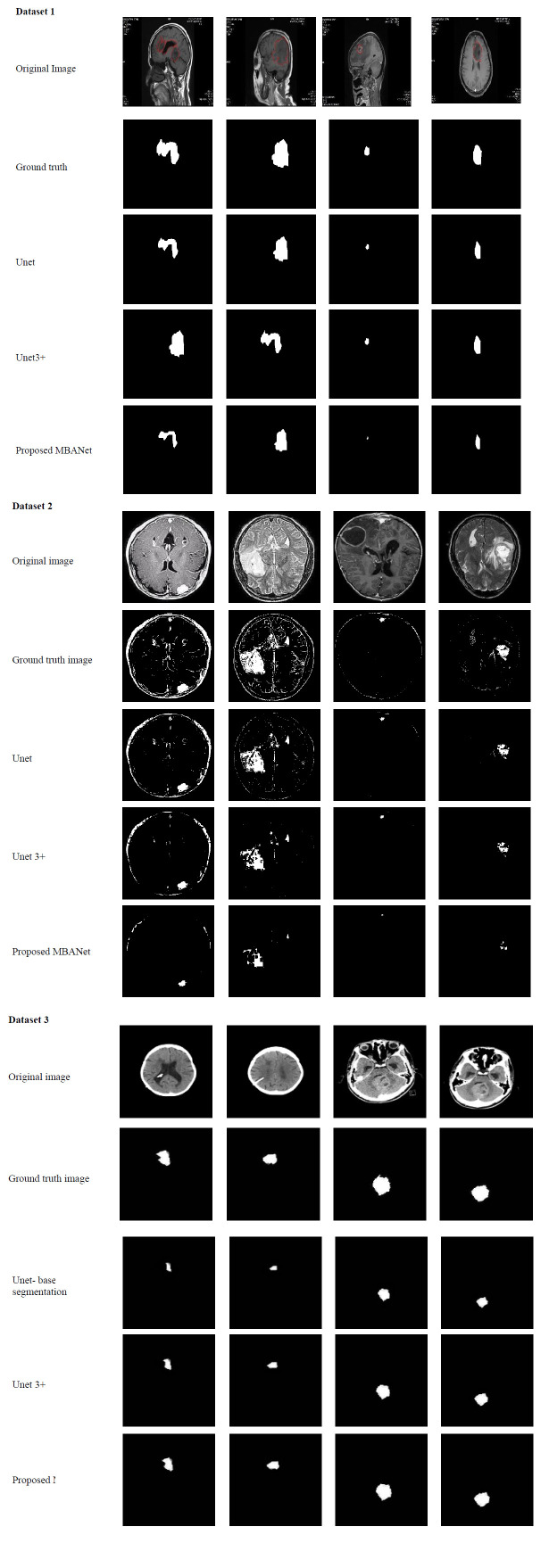
Segmented outcomes from the MBANet model.

**Fig. (5):**
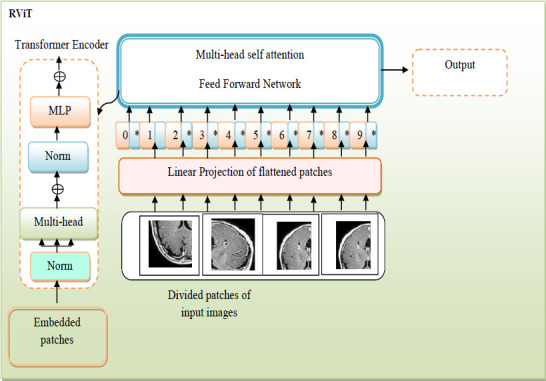
Diagrammatic illustration of the RViT model.

**Fig. (6):**
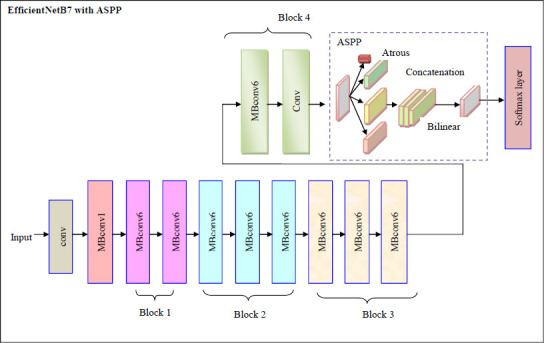
Diagrammatic representation of EfficientNetB7 with ASPP.

**Fig. (7):**
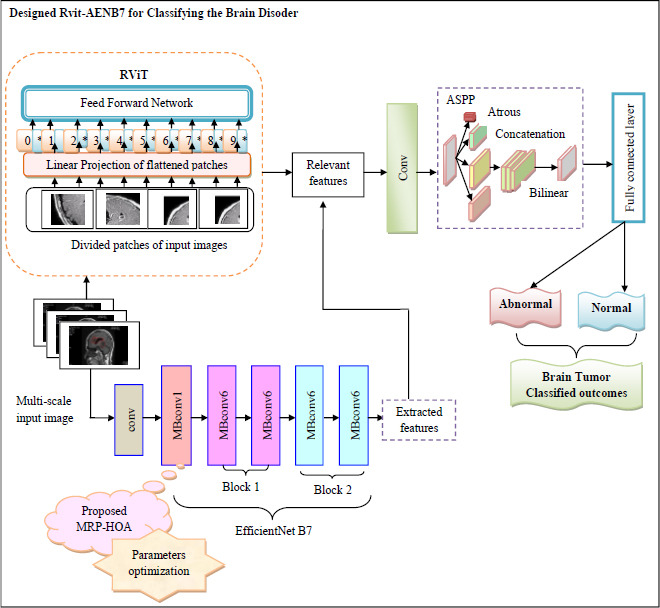
Diagrammatic representation of RVAEB7-ASPP-based brain tumor classification.

**Fig. (8):**
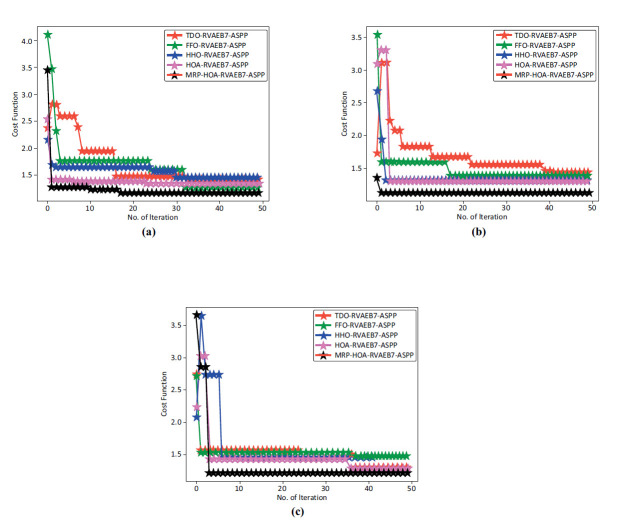
Convergence analysis of proposed brain tumor classification for **(a)** Dataset 1, **(b)** Dataset 2, **(c)** Dataset 3.

**Fig. (9):**
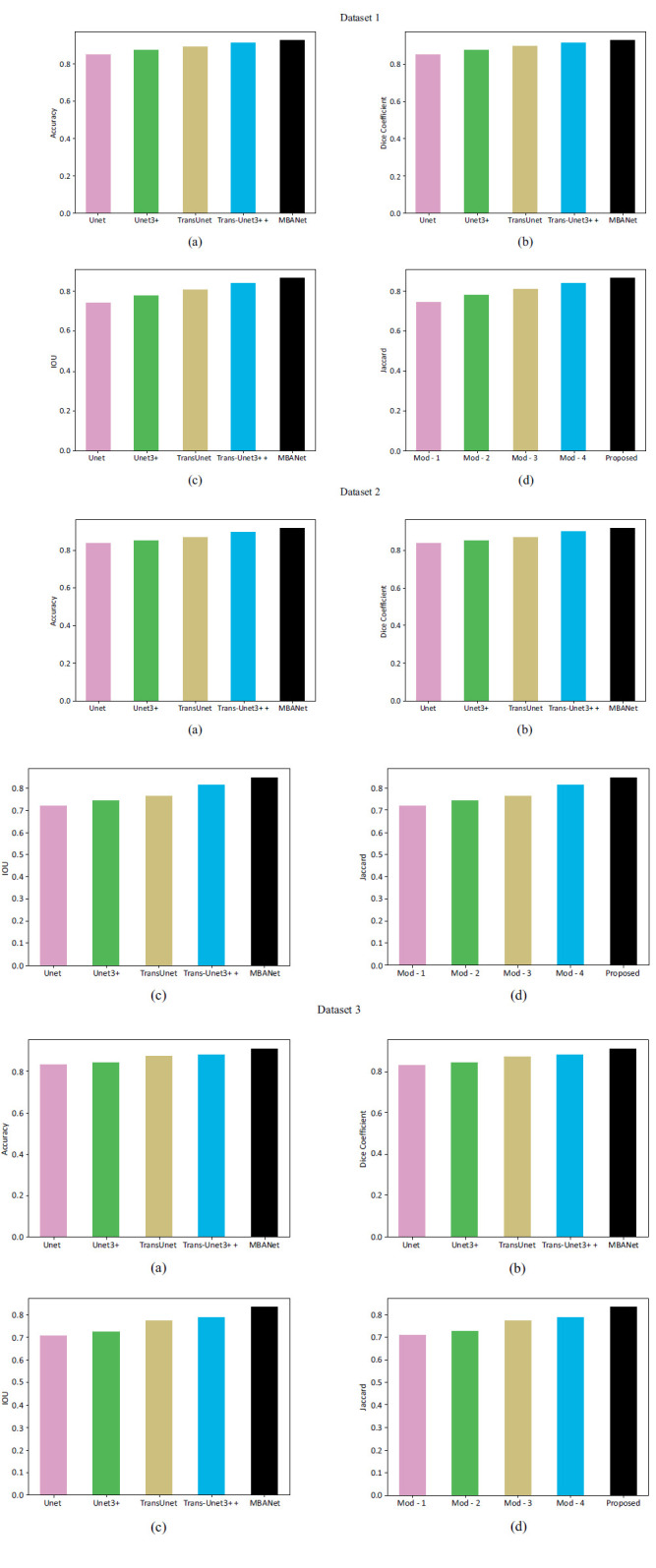
Segmentation analysis of the proposed brain tumor classification model among numerous different techniques regarding. (**a**) accuracy. (**b**) Dice coefficient. (**c**) IOU. (**d**) Jaccard.

**Fig. (10):**
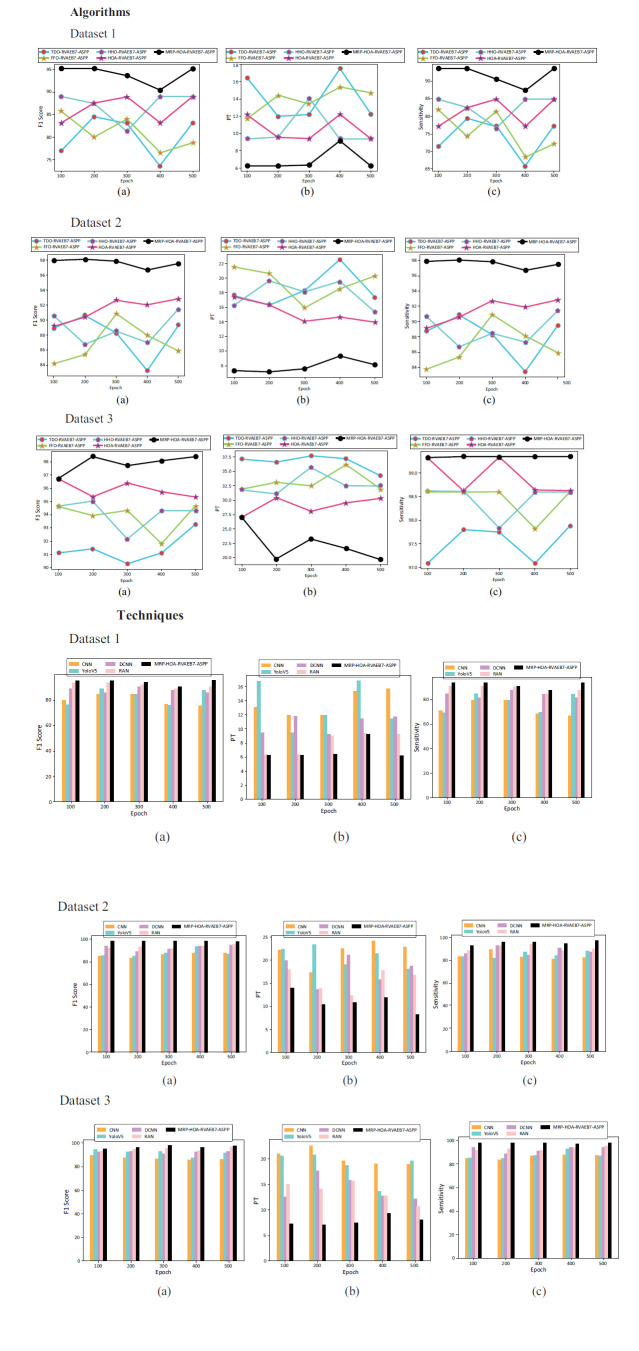
Performance analysis of the proposed brain tumor classification model concerning (**a**) F1-score, (**b**) PT (**c**) Sensitivity.

**Table 1:** Features and challenges of Deep learning-based brain tumor detection and classification models.

**Author Name**	**Methodology**	**Features**	**Challenges**	**Resultant Analysis**
Almufareh *et al*. [18]	Yolo	• The robustness of this method against variations in image quality results in faster and more efficient detection.	• YOLO requires substantial computational resources when trained on large datasets.	• YOLO achieved a recall score of 0.905 for box detection and 0.906 for mask segmentation
Rajendran *et al*. [19]	CNN	• This method exhibits superior performance in image segmentation tasks, achieving high accuracy in identifying and categorizing brain cancers.	• The training process for CNNs necessitates a significant amount of memory and processing capacity, which is not always feasible in clinical applications.	• The obtained accuracy value is 93.4%.
Jabbar *et al.* [20]	Caps-VGGNet	• It is effective at extracting hierarchical features from MRI images. • VGGNet is used in transfer learning, where pre-trained weights are utilized for classification tasks.	• Due to different parameters, VGGNet leads to overfitting issues.	• The obtained accuracy is 97%
Zahoor *et al.* [21]	Res-BRNet	• This model incorporates a data augmentation strategy, which enhances its robustness against variations in MRI images.	• The combination of multiple operations and the need for extensive training data leads to increased computational requirements.	• The proposed model achieved an accuracy of 98.1%.
Khan *et al*. [22]	CNN	• By automating the feature extraction and classification procedures, CNN reduces the likelihood of human errors and enhances the accuracy of brain tumor diagnosis.	• It often functions as a “black box,” making it difficult to classify.	• The obtained accuracy is 83.7%.
Sandhiya *et al*. [23]	DCGAN	• DCGAN is trained to generate cleaner images from noisy input, which leads to improved performance in brain tumor detection.	• The use of synthetic data generated by DCGANs in medical imaging raises ethical issues.	• The obtained precision rate is 96%.
Mehnatkesh *et al.* [24]	ResNet	• This model learns complex features for accurate tumor detection.	• It does not evaluate the nonstructural features of the MRI images.	• This proposed model obtained an average accuracy of 96.6%.
Balamurugan and Gnanamanoharan [25]	DCNN	• This method is successful for determining the structural changes in MRI.	• The computational complexity is high with less accuracy.	• Accuracy is 96.7%

**Table 2:** Hyperparameter details of the proposed model.

**Hyperparameter**	**Values**
Learning Rate	0.0001
Batch Size	32
Number of Epochs	100
Optimizer	Adam
Classification Loss Function	Categorical Cross-Entropy
MRP-HOA Population Size	50
MRP-HOA Number of Iterations	100
Input Image Size	512×512 pixels
Population size	10

**Table 3:** Statistical analysis of the proposed model.

TERMS	TDO-RVAEB7-ASPP [28]	FFO-RVAEB7-ASPP [29]	HHO-RVAEB7-ASPP [30]	HOA-RVAEB7-ASPP [27]	MRP-HOA-RVAEB7-ASPP
**Using Dataset 1**					
Worst	2.821608	4.113312	2.17468	2.549394	3.460062
STD	0.441883	0.505561	0.129819	0.167535	0.31974
Median	1.46688	1.601679	1.610695	1.344542	1.168548
Mean	1.699895	1.655557	1.575547	1.391731	1.243477
Best	1.384623	1.271414	1.449322	1.344542	1.168548
**Using Dataset 2**					
Median	1.56282	1.386905	1.327052	1.306656	1.129438
Worst	3.121558	3.534419	2.675426	3.299299	1.362519
STD	0.338688	0.30676	0.206071	0.457535	0.032631
Mean	1.697512	1.496481	1.366389	1.422094	1.1341
Best	1.447486	1.386905	1.327052	1.306656	1.129438
**Using Dataset 3**					
Median	1.512248	1.51736	1.428442	1.422029	1.208393
Best	1.292227	1.458026	1.263654	1.266849	1.208393
STD	0.209156	0.174711	0.480778	0.34853	0.464988
Mean	1.505734	1.525077	1.565071	1.458013	1.323468
Worst	2.748587	2.733868	3.654935	3.024763	3.666422

**Table 4:** K-fold value evaluation of the proposed model on Dataset 1.

TERMS	TDO-RVAEB7-ASPP [28]	FFO-RVAEB7-ASPP [29]	HHO-RVAEB7-ASPP [30]	HOA-RVAEB7-ASPP [27]	MRP-HOA-RVAEB7-ASPP
**K-fold value is 1**					
Accuracy	88	87	88	90	94
Sensitivity	76.4706	72.973	76.4706	79.4118	87.5
Specificity	93.9394	95.2381	93.9394	95.4546	97.0588
Precision	86.6667	90	86.6667	90	93.3333
FPR	6.06061	4.76191	6.06061	4.54546	2.94118
**K-fold value is 2**					
Accuracy	91	86	93	93	97
Sensitivity	81.8182	72.2222	84.8485	84.8485	93.5484
Specificity	95.5224	93.75	97.0149	97.0149	98.5507
Precision	90	86.6667	93.3333	93.3333	96.6667
FPR	4.47761	6.25	2.98508	2.98508	1.44928
**Among Techniques**					
	CNN [19]	YoloV5 [18]	DCNN [25]	RAN [31]	MRP-HOA-RVAEB7-ASPP
**K-fold value is 1**					
Accuracy	91	89	89	94	94
Sensitivity	81.8182	77.1429	77.1429	87.5	87.5
Specificity	95.5224	95.3846	95.3846	97.0588	97.0588
Precision	90	90	90	93.3333	93.3333
FPR	4.47761	4.61539	4.61539	2.94118	2.94118
**K-fold value as 2**					
Accuracy	84	93	90	94	97
Sensitivity	69.4444	84.8485	79.4118	87.5	93.5484
Specificity	92.1875	97.0149	95.4546	97.0588	98.5507
Precision	83.3333	93.3333	90	93.3333	96.6667
FPR	7.8125	2.98508	4.54546	2.94118	1.44928

**Table 5:** K-fold value evaluation of the proposed model on Dataset 2.

**Among Algorithms**					
TERMS	TDO-RVAEB7-ASPP [28]	FFO-RVAEB7-ASPP [29]	HHO-RVAEB7-ASPP [30]	HOA-RVAEB7-ASPP [27]	MRP-HOA-RVAEB7-ASPP
**K-fold value as 1**					
Accuracy	86.83333	90.2	88.2	88.6	98.26667
Sensitivity	86.85791	90.25367	88.0984	88.75502	98.26667
Specificity	86.80879	90.14647	88.30214	88.44622	98.26667
Precision	86.8	90.13333	88.33333	88.4	98.26667
FPR	13.19121	9.853529	11.69786	11.55378	1.733333
**K-fold value as 2**					
Accuracy	89.46667	92.73333	89.96667	95.3	97.4
Sensitivity	89.46667	92.84759	89.99333	95.33022	97.46328
Specificity	89.46667	92.61968	89.94004	95.26982	97.33688
Precision	89.46667	92.6	89.93333	95.26667	97.33333
FPR	10.53333	7.380319	10.05996	4.73018	2.663116
**Among Techniques**					
	CNN [19]	YoloV5 [18]	DCNN [25]	RAN [31]	MRP-HOA-RVAEB7-ASPP
**K-fold value as 1**					
Accuracy	87.06667	90.46667	91.63333	95.56667	98.26667
Sensitivity	87.06667	90.46667	91.77258	95.53631	98.26667
Specificity	87.06667	90.46667	91.49502	95.59706	98.26667
Precision	87.06667	90.46667	91.46667	95.6	98.26667
FPR	12.93333	9.533333	8.504983	4.402935	1.733333
**K-fold value as 2**					
Accuracy	89.33333	90.16667	89.76667	91.83333	97.4
Sensitivity	89.22872	90.13991	90.00671	91.74983	97.46328
Specificity	89.4385	90.19346	89.52949	91.91717	97.33688
Precision	89.46667	90.2	89.46667	91.93333	97.33333
FPR	10.5615	9.806538	10.47051	8.082832	2.663116

**Table 6:** Dice score-based quantitative analysis of the recommended segmentation model over the baseline approaches.

**Baseline models**	**Dice Score (%)**
U-Net	87.3
Unet3+	88.9
TransUnet	89.6
TransUnet3+	90.7
MBANet (Proposed)	91.4

**Table 7:** Computational cost of the designed model in terms of various parameters.

**Models**	**FLOPs (G)**	**Training Time (hrs)**	**Inference Time (sec)**	**GPU Memory Required (GB)**	**Model Size (MB)**	**Hardware Used**
CNN [19]	6.1	2.4	0.03	4	28	NVIDIA RTX 2060, 16 GB RAM
YoloV5 [18]	17.2	5.6	0.021	6	45	NVIDIA RTX 3060, 32 GB RAM
DCNN [25]	20.3	6.8	0.045	6	34	NVIDIA RTX 3060, 32 GB RAM
RAN [31]	83.7	12.3	0.11	10	93	NVIDIA RTX 3090, 64 GB RAM
RVAEB7-ASPP (Proposed)	42.9	9.4	0.062	8	48	NVIDIA RTX 3080, 32 GB RAM

**Table 8:** Clinical validation of the designed brain tumor detection and classification model.

**Metric**	**Values**
Number of Radiologists Participated	3
Inter-observer Agreement (Radiologist vs AI)	0.86 (Cohen’s Kappa)
Time Saved per Diagnosis	32% reduction
Diagnostic Accuracy (Real-World Dataset)	94.80%
Radiologist Confidence Improvement	+18% (survey-based)
Misclassification Rate (Compared to Human)	3.20%

## Data Availability

The data and supportive information are available within the article.

## LIST OF ABBREVIATIONS

MRI: Magnetic Resonance Images
RVAEB7-ASPP: Region Vision Transformer-based Adaptive EfficientNetB7 with Atrous Spatial Pyramid Pooling
MRP-HOA: Modified Random Parameter-based Hippopotamus Optimization Algorithm
CNNs: Convolutional Neural Networks
MBANet: Multi-Scale Attention Network
RVAEB7-HOA: Region Vision Transformer-based Adaptive EfficientNetB7- Hippopotamus Optimization Algorithm
